# Green Tea Catechins: Nature’s Way of Preventing and Treating Cancer

**DOI:** 10.3390/ijms231810713

**Published:** 2022-09-14

**Authors:** Mohd Farhan

**Affiliations:** Department of Basic Sciences, Preparatory Year Deanship, King Faisal University, Al-Ahsa 31982, Saudi Arabia; mfarhan@kfu.edu.sa

**Keywords:** green tea catechins, EGCG, anticancer, cancer prevention, cancer therapy

## Abstract

Green tea’s (Camellia sinensis) anticancer and anti-inflammatory effects are well-known. Catechins are the most effective antioxidants among the physiologically active compounds found in Camellia sinesis. Recent research demonstrates that the number of hydroxyl groups and the presence of specific structural groups have a substantial impact on the antioxidant activity of catechins. Unfermented green tea is the finest source of these chemicals. Catechins have the ability to effectively neutralize reactive oxygen species. The catechin derivatives of green tea include epicatechin (EC), epigallocatechin (EGC), epicatechin gallate (ECG) and epigallocatechin gallate (EGCG). EGCG has the greatest anti-inflammatory and anticancer potential. Notably, catechins in green tea have been explored for their ability to prevent a variety of cancers. Literature evidence, based on epidemiological and laboratory studies, indicates that green tea catechins have certain properties that can serve as the basis for their consideration as lead molecules in the synthesis of novel anticancer drugs and for further exploration of their role as pharmacologically active natural adjuvants to standard chemotherapeutics. The various sections of the article will focus on how catechins affect the survival, proliferation, invasion, angiogenesis, and metastasis of tumors by modulating cellular pathways.

## 1. Introduction

Camellia sinensis, more often known as tea, is one of the world’s oldest and most consumed beverages. The primary factors that determine the classification of green tea are the history of the production of green tea leaf processing, the location of its origin, and the type of soil on which the tea bushes have grown. The most important producers of green tea are China, Taiwan and Japan. The methodology that is used to produce green tea and black tea is the primary factor that differentiates the two types of tea [1,2,3,4]. There are numerous varieties of green tea, each of which is distinguished from others by the flavor it imparts and the antioxidant capacity it possesses. The methodology involved in tea extraction has a significant weightage on the amount of antioxidant potential that green tea possesses. When compared to black tea, the amount of catechins found in green tea is significantly higher. During the fermentation process, catechins can oxidize into theaflavins, which leads to the occurrence of this effect. In addition, it is essential to be aware of the fact that the concentration of catechins in tea directly correlates to the level of antioxidant activity it possesses. The amount of polyphenolic chemicals, such as catechins, that are produced by Camellia sinensis is directly proportional to the environmental and agricultural circumstances under which it is grown. It is important to note that the antioxidant activity of a green tea infusion rises along with the temperature [5,6], which is an interesting phenomenon. The proportion of green tea’s total content changes based on a range of environmental factors, such as the growing circumstances, the soil, the climate, and other external factors such as light, geography, microorganisms, and temperature [7]. Included in the catechins (flavan-3-ol) family of flavonoids that are found in tea are the following compounds: (−)-epigallocatechin gallate (EGCG), (−)-epicatechin gallate (ECG), (−)-epigallocatechin (EGC), and (−)-epicatechin (EC). One of the most widespread and structurally varied classes of polyphenols is called flavonoids. Strong antioxidant activities are possessed by the molecules as a result of the presence of a large number of hydroxyl groups [1,2]. There are more than 10 different families of chemicals that are found in green tea’s chemical composition. The primary components include catechins, phenolic acids, polyphenolic compounds, amino acids, proteins, and lipids [8,9,10,11,12,13]. Green tea that has not been fermented is notable for being the best source of catechins. The antioxidant capabilities of green tea leaves can vary widely, depending on factors such as their variety and place of origin [13,14,15,16]. Catechins are found naturally in a variety of foods and beverages, including black tea, coffee, berries, grapes, and wine. It is recommended to incorporate foodstuff specifically containing catechins in one’s diet on a daily basis [17] because of the multiple qualities of catechins that are beneficial to one’s health. The catechin group is believed to have the most important impact due to its chemopreventive, anti-inflammatory, and antioxidant properties [13,14,15,16,17,18]. Catechins have the antioxidant properties of scavenging reactive oxygen species, decreasing the generation of free radicals, and preventing lipid peroxidation. These are the fundamental roles that catechins play in plants. According to the research that has been published so far, the antioxidant activity of catechins found in green tea and their significant impact on the prevention of diseases associated with modern civilization are largely determined by the presence of structural groups in the molecules, in addition to the number of hydroxyl groups [3,4,19,20]. Evidence suggests that green tea may help reduce the risk of numerous types of cancer, including those of the esophagus, lung, prostate, stomach, breast, pancreas, intestinal tract, and bladder [14,15,16,17].

**Figure 1:**
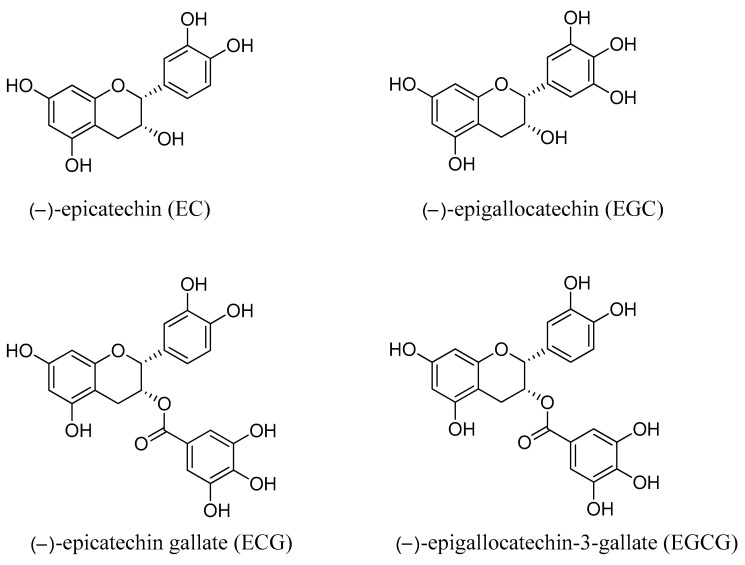
Chemical structure of green tea catechins.

In this article, the following topics are discussed with reference to green tea catechins: (i) chemical composition, (ii) health advantages, (iii) mechanism of action, (iv) anticancer potential, and (v) anticancer signalling pathways. The chemical structures of the most abundant catechins in green tea are depicted in Figure 1.

## 2. Chemical Composition of Green Tea Catechins

Green tea has a protein content of about 15–20%, which includes amino acids such as L-theanine [10], tryptophan, tyrosine, leucine, threonine, 5-N-ethylglutamine, lysine, glutamic acid, serine, glycine, valine, aspartic acid, and arginine. The protein content of green tea can be broken down into two categories: complete proteins and incomplete proteins. Green tea may also contain trace elements, including magnesium, chromium, manganese, calcium, copper, zinc, iron, selenium, salt, cobalt, or nickel, as well as carbohydrates, including glucose, cellulose, and sucrose [9,10,11,12,13,14,15,16,17,18,21,22]. In addition, green tea is abundant in sterols and lipids, including linoleic and linolenic acid, as well as some vitamins (A, B, C, and E); the highest concentrations of these nutrients can be found in Gyokuro tea (approximately 10 mg) and Sencha tea (4 mg). Vitamin K is present in only trace amounts. Matcha tea is the sole known source of vitamin A. Phosphorus, fluorine, and iodine are some of the macro-elements that can be obtained from drinking green tea. Additionally, green tea is an excellent source of phosphorus. Another characteristic of green tea is the presence of the diphenylpropanoid skeleton, which has the chemical formula C_6_C_3_C_6_ [10,11,12]. In addition, green tea has a high concentration of xanthine bases, such as theophylline and caffeine [21], as well as a variety of pigments, including chlorophyll and carotenoids. It is important to note that the chemical makeup of green tea also consists of phenolic acids, which can include gallic acid, as well as volatile chemicals, which can include alcohols, esters, hydrocarbons, and aldehydes. Phenolic acids, which include proanthocyanidins and also gallic acid esters with monosaccharides, have a significant influence on the characteristics of green tea infusion. These phenolic acids also play a role in the formation of catechins. The collection of chemicals known as polyphenols also includes flavonoids, flavandiols, and flavols, in addition to the phenolic acids that were just discussed. According to the data that are now available, these chemicals may account for as much as 30–35% of green tea’s dry matter. Catechins are the most common type of flavonoids found in green tea. When compared to black or Oolong tea, the amount of catechins found in green tea is significantly higher. As mentioned above, the catechins group primarily consists of EGCG, ECG, EGC, and EC [10,11,12,13]. When considering the antioxidant activity of catechins, it is crucial to take into account both the total amount of hydroxyl groups as well as the distribution of those hydroxyl groups [16,19,20]. When compared with catechins that have a pyrogallol group, those that contain a catechol group are found to have a reduced antioxidant potential. However, the ability of catechins to act as antioxidants is dependent not only on their chemical composition but also on the circumstances in the surrounding environment [10,11,12,13,14,15,16,17,18]. The various chemical components of green tea each have a fundamentally distinct impact on the development of a certain form of cancer. According to the evidence that is currently available, ascorbic acid, arginine, proline, lysine, and EGCG have all been found to have a beneficial effect on the suppression of tumor growth [12,13,14,15,16,17]. The amount of EGCG found in various foods is outlined in Table 1.

## 3. Green Tea Catechins Health Beneficial Properties

The manufacturing of green tea does not involve fermentation, in comparison to the making of black tea, which involves the transformation of Oolong by fermentation. Enzymes have an effect on the catechin polyphenols that are present in the leaves of the tea bush, which leads to the process of full fermentation, which is necessary for the production of black tea [14]. Given the fact that there are numerous ways by which the leaves of Camellia sinensis are processed, each kind of tea has its own unique effect, in addition to other biologically active components and qualities that are beneficial to one’s health [14,15].

Green tea contains a variety of beneficial polyphenols, in particular flavonols and flavanols, which are responsible for the tea’s positive effects on health. Experiments, both in vivo and in vitro, as well as clinical investigations, have shown that they have antioxidant, anti-inflammatory, and cardiovascular properties. Catechins are the predominant type of polyphenol found in green tea. The antioxidant properties of catechins are due to their ability to chelate metal ions (specifically copper ions) in redox reactions as well as to neutralize free radicals of oxygen. A number of human epidemiological and clinical studies on tea have revealed evidence for its chemopreventive effects, which have been validated by cell-based and animal investigations. Furthermore, specific chemical mechanisms underlying the action mechanism of EGCG and other catechins have been postulated. One of the most appealing methods is the involvement of reactive oxygen species (ROS). EGCG is known to have both antioxidant and pro-oxidant properties in regard to ROS [19,20]. The polyphenols found in green tea leaves have been shown in a number of scientific studies to have anti-tumor properties. These properties include the inhibition of cell division as well as the induction of phase II antioxidant enzymes. Some examples of these enzymes include superoxide dismutase, glutathione-S-transferase, glutathione peroxidase, and glutathione reductase. According to the literature, drinking green tea for a period of four months at a rate of four glasses per day can reduce the amount of 8-hydroxydeoxyguanosine found in the urine [30]. Numerous scientific studies in the fields of prostate, pancreatic, breast, and stomach cancers [14,15,16] have confirmed that the effects of green tea polyphenols on inhibiting the growth of cancer cells and reducing the risk of cancer can reduce the chances of developing cancer [14]. Green tea polyphenols have been shown to inhibit the growth of cancer cells [31]. Green tea may enhance chemotherapeutic as well as preventative benefits; however, it cannot replace the treatment provided by pharmaceuticals. This is an important point to emphasize. Notably, polyphenols, which include catechins, have the ability to trigger the death of cancer cells without damaging the surrounding healthy cells [9,14,17].

### 3.1. Action Mechanism of Green Tea Catechins

EGCG is the catechin derivative that has received the most attention to date. The amount of catechins that are present in a cup of green tea is mostly determined by the type of green tea, how it was grown, and how the leaves were processed, as well as how long it was brewed and at what temperature. According to a number of studies [9,10,11,12,13], catechins are at their most stable in an environment with a pH varying between 4 to 6. The research that is currently available suggests that catechins have a number of health benefits, including those that are anticancer [9], anti-inflammatory [32], anti-microbial [33,34,35], anti-viral [33,34,35], anti-diabetic [33,34,35], and cardiovascular [33,34,35]. It is important to underline the good effects that they have not only on bacteria but also on viruses, fungi, and prions [1]. Additionally, catechins have the ability to chelate metal ions, specifically copper ions [19,20,36]. Copper levels are known to be greatly raised in cancer cells [37]; in a study where copper was orally administered to rats in an attempt to imitate similarly high copper concentrations in normal lymphocytes [38], it was discovered that oral administration of copper to rats increased serum and lymphocyte copper levels. When these lymphocytes with an excess of copper were treated with EGCG, the DNA damage was substantially enhanced. Moreover, treatment of isolated rat cells with EGCG at increasing concentrations led to a steady increase in DNA damage. However, this increase in cellular DNA breakage was more pronounced in the lymphocytes of copper-treated rats than in those of untreated rats. This suggests that copper is involved in the EGCG-induced cellular DNA breakage.

The specific chemical structure of polyphenols found in green tea (the presence of a minimum of five hydroxyl groups) has a substantial impact on antioxidant capacity [19,20,39,40,41,42]. This is because green tea contains at least five hydroxyl groups. The di/tri-hydroxy structure of the B and D rings [39] as well as the meta-5,7-dihydroxy group at the A ring [1,43,44] make it possible for chelation of transition metal ions to occur [19,20]. For synthesizing any novel anticancer molecule(s) based on the structure of catechins, (i) the molecule should be of epicatechin type; (ii) it should have as many galloyl moieties as feasible as this would increase the number of hydroxyls capable of binding to DNA and Cu(II) and reducing it to Cu(I) [19]. Nevertheless, in particular circumstances, they might have effects that are pro-oxidative [19,20,45]. The regulation of catechins within an intracellular pool of nitro-oxidative stress is the primary mechanism by which these compounds exert their anticancer effects [46]. Therefore, polyphenolic chemicals, which bring health-promoting benefits for the body, can also result in the opposite consequences if very high dosages of catechins are utilized [47]. This is because catechins are a type of antioxidant. As a consequence, this leads to the induction of pro-oxidative stress as well as oxygen damage to the constituents of the cell. In addition, polyphenols have an effect that is similar to that of oxidizing enzymes such as tyrosinase and peroxidase when these enzymes are present in the body. In addition, there is a close connection between the processes of inflammation and the pro-oxidative action [47]. Catechins have qualities that are both antioxidant and pro-oxidant, and both of these properties are dependent on the same variables inside the molecule, such as the quantity of hydroxyl groups present [19]. During the process of oxidation of polyphenols, reactive oxygen species and electrophilic quinones cause damage to the molecules of cells [48,49]. This damage is caused by polyphenols. This factor plays a critical role in the etiopathogenesis of degenerative disorders as well as a process that can cause cancer [47].

Furthermore, catechins and other active compounds obtained from green tea have the ability to repair DNA damage brought on by exposure to UV-B radiation. Catechins are particularly effective at doing this. The data that are currently available suggest that the active compounds in green tea have a high level of efficiency in protecting the skin from the harmful effects of UV radiation [21].

### 3.2. Green Tea Catechins’ Anticancer Potential: In Vitro and In Vivo Studies

EGCG, which is known as the major polyphenol in green tea and contains eight different hydroxyl groups, is the most powerful bioactive component that green tea possesses [21,50,51]. In a prior study using an animal model of prostate cancer [50], the effect of catechins in inducing cell death was demonstrated and validated. There has been a lot of research done to confirm that EGCG can induce apoptosis and stop the cell cycle. One example of this would be in HCT-116 cells, which are used to study colon cancer [52,53]. It is widely recognized that the suppression of metalloproteinase activity by EGCG is the primary anticancer mechanism that it possesses. This idea has been given acceptance by a study that found a reduction in the number of metastases caused by prostate cancer following oral supplementation with catechins from green tea [46]. In an animal model of melanoma, catechols found in green tea were shown to limit the spread of cancer to the lungs [51]. In addition to this, there was revealed to be a positive correlation between the use of green tea and the risk of developing bladder cancer. There are additional studies that confirm the preventative role of green tea against colorectal adenomas after ingesting ten cups of green tea that are each 150 milliliters in size [51]. As discussed previously, copper levels are well elevated during the progression of cancer [37]. In another set of experiments, diethyl nitrosamine (DEN) was administered to induce hepatocellular carcinoma (HCC) in rats in order to confirm the role of copper and EGCG in oxidative DNA breakage [54]. After DEN therapy, there was a steady increase in intracellular copper. Upon treatment of HCC cells to EGCG, ROS-mediated DNA damage was also observed. Neocuproine, a copper chelator that permeates cell membranes, prevented the EGCG-mediated DNA breakage in these cells. A membrane-impermeable copper chelator, bathocuproine, was shown to be ineffective. Similarly, a specific iron chelator (desferoxamine mesylate) and zinc chelator (histidine) failed to suppress EGCG-mediated DNA breakage in cells, supporting the involvement of endogenous copper in cell death.

Breast cancer is one of the most common cancers found in females around the world. Research into the effects of green tea catechin derivatives, such as chemopreventive as well as synergistic effects along with chemotherapy, has been conducted on breast cancer cells in multiple trials in both the laboratory and clinically [51,55]. As a consequence, these catechins may have potential chemopreventive properties. At the moment, lung cancer is the most prevalent kind of cancer in the entire world. In the case of animal models (mice), it has been demonstrated that taking EGCG in oral supplement form has an effect on H1299 human non-small cell lung cancer xenografts. The findings of this research suggest not only an increase in the death of cancer cells by apoptosis but also a suppression of the growth of tumors in lung cancer [51,55,56]. In addition, the administration of EGCG in the form of an oral supplement promoted the production of reactive oxygen species in the mitochondria of lung cancer cells [51,55,56]. This may have occurred as a result of the low number of antioxidant enzymes present in these cells. It has been demonstrated that the addition of catechins generated from green tea to the medium used in cell culture causes an increase in the degree of oxidative stress, which ultimately results in apoptosis [50,57]. As a result of this, numerous in vivo studies have been conducted to assess the effect of the amount of green tea consumed on the decrease in the incidence of malignant tumors, such as colorectal cancer, stomach cancer, liver cancer, or lung cancer [47,55]. These findings pertain to consuming greater than ten cups of green tea infusions on a daily basis [58]. On the other hand, one study found that drinking five to nine cups of green tea infusions per day was associated with an increased risk of developing bladder cancer [59]. The favorable anticancer impact of EGCG, the major catechin found in green tea, was further confirmed by inhibition of osteoblastic differentiation [60]. Additionally, cancer stem cells have been used in research to investigate EGCG’s possible anticancer effects [46]. Stem cells, also known as precursor cells, are defined by the ability to proliferate, also known as self-renew, and maintain a constant, unchanging number of cells. In addition, stem cells have the ability to specialize as the right cell type when necessary. Notably, both the extract of green tea and EGCG have been shown to limit the development of cells in these cellular and animal models [61,62]. Studies both in vivo and in vitro have shown that cancer stem cells are responsible for the renewal of cancer as well as the spread of the disease [46]. According to the data that are currently available, tumor stem cells are able to circumvent the epithelial-mesenchymal transition during the process of metastasis [46]. The cancer cells are able to travel toward the blood arteries as a result of this procedure. When compared to a cancer cell, cancer stem cells display a significantly better capacity for oncogenesis [46]. This is an important finding that warrants attention. After conducting an in-depth analysis of the existing scientific study on the effect of green tea catechins on cancer stem cells, some literature was found suggesting the influence that Matcha green tea catechins have on the oxidative phosphorylation of MCF-7 breast cancer stem cells [46]. Additionally, treatment of MCF-7 breast cancer cells with an extract of Matcha green tea creates a considerable effect on the IL-8 pathway, which is implicated in the proliferation and angiogenesis of migratory cancer cells [46]. This therapy also has an influence on the control of the cell cycle. Multiple studies have demonstrated that EGCG induces apoptosis in various types of cancer cells by activating apoptosis-related molecules. Some of them have been summarized below in Table 2 and Table 3.

## 4. Signaling Pathways in Green Tea Catechins Anticancer Activity

The cell signaling pathways that are crucial for maintaining the equilibrium between cell proliferation and cell death have emerged as rational targets for anticancer therapies in recent years. As discussed, the catechins procured from green tea, specifically the most powerful EGCG, are able to trigger apoptosis in a variety of cancer models. In particular, it is able to trigger apoptotic pathways that are both intrinsic (using the mitochondria) and extrinsic (involving the death receptor) [76]. After being treated with catechols from green tea, cells exhibited several hallmarks of the apoptotic process, including nuclear condensation, caspase-3-activation, and cleavage of poly(ADP)ribose polymerase [77]. In addition, the activation of BAX, depolarization of mitochondrial membranes, and release of *cytochrome c* into the cytosol are all components of the anticancer mechanism that EGCG possesses [78]. The primary pathways utilized in the process of controlling cell proliferation are known as the induction of cell cycle arrest and apoptosis. As a matter of fact, catechols found in green tea have been shown to regulate both the G1/S transition and the G2/M transition in addition to preventing a rise in the number of cells and in DNA synthesis [76]. Most importantly, EGCG causes apoptosis (programmed cell death) and inhibition in the cell cycle in many cancer cells, while it does not have any effect on normal cells [12]. Research has shown that EGCG has the ability to directly block cyclin-dependent kinases [44], which is the primary event in the progression of the cell cycle. Additionally, EGCG lowers the expression of cyclin D1 and increases the phosphorylation of retinoblastoma [76]. This occurs despite the fact that it enhances the expression of p21 and p27. The molecular signaling pathways that are regulated by green tea catechols and that result in their pro-apoptotic and anti-proliferative effects include, among other things, suppression of nuclear factor kappa-B (NF-kB), which is the essential oxidative stress-sensitive transcription factor [14,51]. Inflammation, cell proliferation, and the death of cancer cells are just a few of the biological responses that are regulated by the transcription factor NF-kB, which plays an essential part in this process. In addition, endothelial nitric oxide synthase (eNOS) is stimulated by the catechins that are found in green tea, particularly the primary catechin known as EGCG [61,79].

The inhibition of mitogen-activated protein kinases (MAPKs), such as ERK, JNK, and p38, in the presence of EGCG, has been shown to have a favorable role in a wide variety of pathophysiological processes, including cell proliferation, differentiation, and the death of cancer cells [47,56,64,78]. In addition, it is well established that exposure to EGCG inhibits the action of tumor necrosis factor (TNF-α), which in turn causes cancer cells to undergo the process of apoptosis [60]. The suppression of the epidermal growth factor receptor (EGFR)-mediated signal transduction pathway is yet another event in the molecular signaling cascade that is affected by the catechols found in green tea. The epidermal growth factor receptor (EGFR) is a glycoprotein that is found in the plasma membrane. It contains an extracellular ligand-binding domain, a single transmembrane region, and an intracellular domain that possesses intrinsic tyrosine kinase activity. When tumor cells have an excessive amount of EGFR expression, they take on a neoplastic phenotype. Specifically, EGCG prevents the activation of the epidermal growth factor receptor (EGFR), the human epidermal growth factor receptor 2 (HER2), and a number of other signaling pathways that are downstream in colon cancer cell lines [12,51]. It is important to note that the molecular signaling pathways of green tea catechols involve the additional suppression of insulin-like growth factor-I (IGF I)-mediated signal transduction [12]. Catechins found in green tea have been shown to considerably lower levels of the protein IGF-I in animal models of prostate cancer [80]. The data from the published research suggest that polyphenols produced from green tea exert their anticancer effect due to modifications of histones, micro-RNA, and DNA methylation [58].

EGCG plays a leading role in the suppression or uncontrolled angiogenesis process, inhibiting the pro-angiogenic VEGF factors. High concentrations of EGCG have been shown to inhibit the production of VEGF in breast cancer cell lines [81]. It has been reported that EGCG inhibits the activity of VEGFR-2 [82]. Additionally, EGCG suppresses the activation of HIF-1 and NF-kB, as well as the expression of VEGF, hence inhibiting tumor angiogenesis and breast cancer growth. In addition, the results demonstrated that EGCG administration significantly decreased tumor weight compared to the control group and tumor VEGF expression [83]. By reducing the constitutive activation of Stat3 and NF-kB in cancer cells, EGCG decreases the synthesis of VEGF. EGCG targets the phosphoinositide-3-kinase (PI3K) and Akt/Protein Kinase B (PI3K/Akt) pathway. Despite the fact that the molecular mechanisms behind the combined action of autophagy and apoptosis have not yet been explained, a few studies have investigated the signaling pathways altered by EGCG. It has been demonstrated that EGCG induces apoptosis via the PI3K/Akt pathway [84]. According to Yin et al. [85], both EGCG and the PI3K/Akt inhibitor LY294002 cause enhanced apoptosis in the 5637 cell line, whereas their combination produces the maximum apoptosis induction efficiency. The authors demonstrate that EGCG inhibits cell proliferation by promoting the interaction between autophagy and apoptosis by inactivating the PI3K/Akt pathway (needed for autophagy induction). Similarly, Hsieh et al. demonstrate that EGCG administration lowers Akt phosphorylation in PANC-1 cells, suggesting that the downregulation of p-Akt is related to the development of cytotoxic autophagy [86].

**Figure 2:**
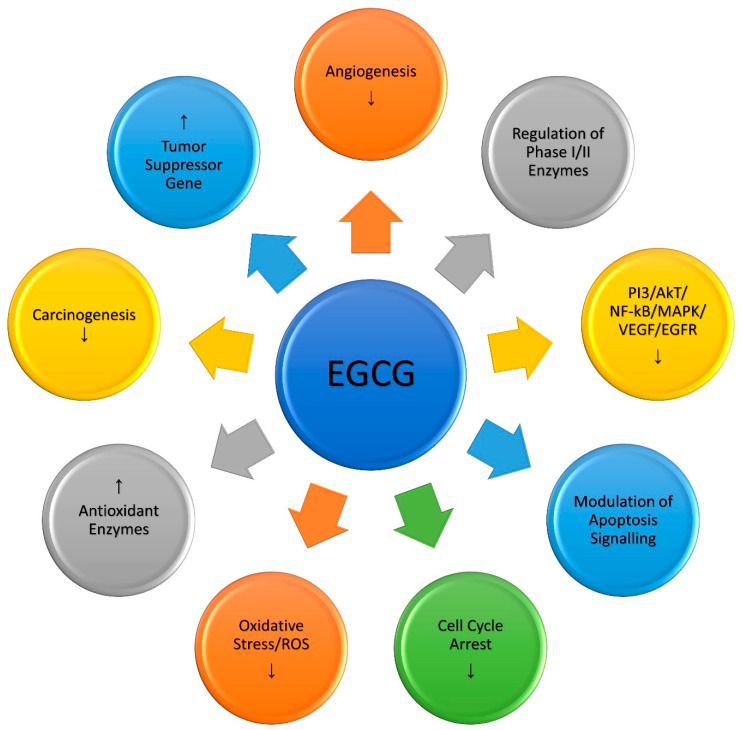
Molecular mechanisms involved in the anticancer activity of EGCG; (↑ up regulation, ↓ down regulation).

Recent research has also demonstrated that copper-enriched mediums make normal breast epithelial cells (MCF-10A) more susceptible to EGCG-mediated growth inhibition. This sensitization also increases the expression of the membrane-bound copper transporters CTR1 and ATP7A [57]. The overexpression led to an increase in copper buildup in such cells [57,87]. siRNA-mediated suppression of the copper transporter CTR1 reduced the sensitivity of these cells to EGCG-mediated cell death. ROS scavengers (superoxide dismutase (SOD) for superoxide anion, catalase for hydrogen peroxide, which can form hydroxyl free radicals, and thiourea for scavenging hydroxyl free radicals) could lessen this DNA breakage. Therefore, it was deduced that the participation of nuclear copper plays a key role in cellular DNA breakage produced by plant polyphenols and that reactive oxygen species play a crucial function in inducing DNA breakage in this reaction. Therefore it may be concluded that copper-induced, non-enzymatic, free radical-mediated DNA cleavage by EGCG may be one of the multiple non-genomic pathways of EGCG-induced selective cancer cell death. The EGCG-induced major molecular pathways involved in anticancer activity are shown below (Figure 2)

## 5. Green Tea Catechins Anticancer Potential: Clinical Studies

Literature data strongly demonstrate that EGCG controls multiple molecular targets and inhibits the pathogenesis of cancer by preventing its initiation, development, and advancement. In addition, human clinical trials are still required to establish the efficacy of EGCG in the treatment of cancer. Multiple human clinical investigations show that EGCG has a role in cancer prevention. For the same purpose, clinical trials are now utilizing Polyphenon E (Poly E), a well-standardized decaffeinated green tea catechin combination that comprises 65% EGCG and EC [88].

In a randomized, placebo-controlled clinical trial of Poly E, 97 men with high-grade prostatic intraepithelial neoplasia and/or atypical small acinar proliferation took 400 mg EGCG per day. The main goal of the study was to compare the total rates of prostate cancer in the two study groups after one year. There were no changes in the number of men who got prostate cancer. This was shown by the fact that there were fewer cases of atypical small acinar proliferation in the Poly E arm (0/26) than in the placebo arm (5/25). On the Poly E arm, the serum prostate-specific antigen level went down. Consuming a standardized, decaffeinated catechin mixture of 400 mg EGCG every day for a year was well tolerated and collected in plasma, but it did not lower the risk of prostate cancer [89].

In another study, 97 men with high-grade prostatic intraepithelial neoplasia and/or atypical small acinar proliferation took Poly E (400 mg EGCG per day). The study was controlled by a placebo and used a randomized clinical trial design. Along with diet, Poly E was given with 200 mg of EGCG. In this trial, a secondary study goal was to compare the total number of treatment-related side effects. It was observed that side effects of grade 3 or higher had happened over the course of one year on the two study arms. The toxicity, medications taken at the same time, and organ function were all checked every month. Men who had high-grade prostatic intraepithelial neoplasia or atypical small acinar proliferation at the start of the study were given a standardized, decaffeinated catechin mixture with 200 mg EGCG twice a day with food for a year. The treatment was well tolerated and did not cause any side effects [90].

A phase II pharmacodynamic prevention trial of Poly E was done on patients before they had surgery for bladder cancer. Patients with bladder tumors were given either Poly E with 800 mg or 1200 mg of EGCG or a placebo for 14 to 28 days before their bladder tumors were removed through the urethra. The levels of EGCG in tissue did not change much between the two Poly E dosage groups combined and the placebo group. However, a dose-response relationship was seen for the levels of EGCG in both normal and cancerous bladder tissue in all three study arms. Additionally, the amount of EGCG in the blood and urine went up, and the amount of clusterin went down in a dose-dependent way. The levels of EGCG in plasma, urine, and bladder tissue were related to the dose, and the levels of tissue biomarkers of proliferation and apoptosis were also affected by the dose [91].

A phase I study was done to find if using EGCG mouthwash with radiation for head and neck cancer was safe and if it worked. In this study, people with head and neck cancer were given EGCG mouthwash at the assigned dosage level, which started at 440 micromols per liter and was given three times a day. This is called a standard 3 plus 3 dose escalation design. Every week, mucosal toxicity, pain from mucositis, and patient satisfaction were noted. The first goal was to see if this green tea compound was safe, and the second goal was to see if it helped relieve the symptoms of mucositis. The highest amount of EGCG that could be used in the mouthwash was 2200 micromol per liter. Burning and nausea were the most common types of toxicities. Mucositis-related pain scores considerably decreased after EGCG administration over time. The addition of this EGCG mouthwash to radiotherapy is feasible without increasing toxicities. The recommended dose for a phase II study was determined to be 1760 micromol per liter, and EGCG administration decreased radiation-induced oral mucosal injury in patients [92].

The tolerability, safety and effectiveness of topical EGCG for radiation dermatitis of breast cancer patients receiving adjuvant radiotherapy were investigated. Breast cancer patients who received radiotherapy to the chest wall after mastectomy were included. This green tea compound was sprayed into the radiation field from the beginning of grade 1 radiation dermatitis for two weeks after finishing radiotherapy. EGCG concentration escalated from 40 to 660 micromol per liter in 7 levels, with 3 to 6 patients in each level. Acute skin redness was noticed in 1 patient and measured to be related to the EGCG treatment at 140 micromols per liter level. Some patients included at this level did not experience toxicity to EGCG. No other reported acute toxicity was related to EGCG. Grade 2 radiation dermatitis was noticed in eight patients during or after radiotherapy. The topical administration of EGCG was well tolerated, and the maximum tolerated dose was not found [93].

A two-stage, single-arm, phase II trial evaluated the safety and efficacy of EGCG enriched tea drink in women with advanced serous or endometrioid ovarian cancer. Participants had FIGO stage III-IV serous or endometrioid ovarian cancer. All participants drank 500 mL of double-brewed green tea every day for 18 months or until recurrence. Here, 18-month recurrence-free survival was the main goal. Only 5 of 16 women were free of recurrence 18 months following complete response in the first stage of the research. The clinical trial was canceled. Six ladies stopped drinking double-brewed green tea before the study’s end. After standard treatment for advanced ovarian cancer, double-brewed green tea does not appear to be a promising maintenance strategy [94].

EGCG was studied in stage III unresectable lung cancer. Radiation was given alongside cisplatin and etoposide. EGCG solution was administered three times a day at six concentration levels following grade II esophagitis. Dose escalation, oesophageal toxicity, and patient-reported discomfort were assessed weekly. EGCG was given in six dosage cohorts. All EGCG dosing tiers lacked dose-limiting toxicities. After radiotherapy, 22 of 24 patients had grade 0/I esophagitis, whereas 2 had degree 2 esophagitis. EGCG oral administration is practical, safe, and efficacious. The phase II recommended concentration of EGCG is 440 micromol/L [95].

## 6. Limitations of Using Green Tea Catechins as Anticancer Agents

Human epidemiological research has shown a link between consuming natural polyphenols and a lower risk of developing cancer. A typical polyphenol, EGCG, has been the subject of much research over the past several years that has examined its positive impact on health. The chemopreventive effect of EGCG depends on how well it is absorbed by the body and how well it interacts with target tissues. However, EGCG is thought to have low lipophilicity, which makes it less likely to pass through membranes, especially the intestinal epithelium [96]. Since it does not have a receptor-mediated transport, it is likely that the permeability of its membrane depends on passive diffusion [97]. Most of the time, a fairly high concentration of catechin is needed for EGCG to be useful as a medicine. For in vitro studies, an effective concentration of EGCG is usually between 1 and 100 mol/L. However, this value is hard to reach in in vivo conditions because the plasma peak of tea catechins is in the sub- or low-micromolar range [98].

Additionally, catechins do not absorb well, are unstable in the digestive tract, and are not very bioavailable. This makes it hard for them to reach the therapeutic target. Therefore, the fact that the biological effects of EGCG from in vitro and in vivo studies are not always the same is often due to the fact that it is not very stable. Under physiological conditions, catechins are quickly metabolized and changed into degradation products or pro-oxidant molecules, no matter how they are given [99,100]. On the other hand, too many catechins may help achieve the right doses of bioactivity, but this is linked to a toxicological response that depends on the dose [101]. Much work is being done to make EGCG more bioavailable and improve its low uptake by cells. Putting EGCG inside hydrophobic nanocarriers has led to some promising results [97,98,102,103].

These systems stop the catechins from breaking down and being metabolized while they are being transported, so there are more catechins in the bloodstream [98].

## 7. Conclusions

Green tea catechins are strong bioactive substances with anticancer properties. By altering crucial cellular processes such as cellular proliferation, differentiation, apoptosis, angiogenesis, and metastasis, they prevent the onset, growth, and progression of cancer. Despite being taken for centuries, green tea has only lately been the subject of in-depth research as a beverage that may help prevent a variety of ailments, including cancer. Numerous investigations have shown that green tea catechins have an impact on numerous biological pathways and that polyphenols potently promote apoptotic cell death and cell-cycle arrest in tumor cells but not in their normal cell counterparts. Multiple animal studies have shown that green tea therapy reduces the incidence and growth of tumors in a variety of organ sites, including the skin, lung, liver, stomach, mammary gland, and colon. Phase I and II clinical trials have recently been carried out to investigate the anticancer properties of green tea in people. Researchers should continue to obtain a better understanding of the pharmacokinetics of tea polyphenols, including absorption, distribution, their function in anticancer responses, metabolism, and molecular mechanisms, through studies focusing on polyphenol EGCG. In order to find more potent, stable, and selective tea polyphenol analogs as possible novel anticancer medicines, work should continue on improving and assessing further analogs of green tea catechins. However, structural modification studies of EGCG have shown promising findings regarding their anticancer properties. Integrating new molecular discoveries into clinical practice is a significant challenge in cancer prevention. To effectively prevent and cure cancer with synthetic EGCG analogs, it is essential to identify more molecular targets or biomarkers for green tea polyphenols. This will also help us better understand the anticancer mechanisms of action.

## Figures and Tables

**Table 1:** Distribution of EGCG in various foodstuffs.

Food Stuff	Content	Reference
Green tea	4.62 mg/100 mL	[23]
Green tea (loose leaf form)	56.5–205.0 mg/g dry tea	[24]
Green tea (bagged leave form)	54.3–153.0 mg/g dry tea	[24]
Green tea (infusions)	117 to 442 mg/L	[25]
Apples, Fuji, raw, with skin	1.93 mg/100 g edible portion	[26]
Blackberries, raw (Rubus spp.)	0.68 mg/100 g edible portion	[26]
Cranberries, raw	0.97 mg/100 g edible portion	[26]
Nuts, pecans	2.30 mg/100 g edible portion	[27]
Tea, black, brewed, prepared with tap water	9.36 mg/100 g edible portion	[27]
Tea, fruit, dry	415.0 mg/100 g edible portion	[27]
Tea, green, brewed	64.0 mg/100 g edible portion	[27]
Tea, green, large leaf, Quingmao, dry leaves	7380 mg/100 g edible portion	[27]
Tea, white, dry leaves	4245 mg/100 g edible portion	[27]
Japanese green tea	18.1–23.1 mg/g	[28]
Long-jing tea	32.9–35.5 mg/g	[28]
Jasmine tea	29.8–31.0 mg/g	[28]
Sen-cha (tea bags)	12.9–23.6 mg/g DM	[29]
Carob flour	109.46 mg/100 g edible portion	[27]

**Table 2:** Cancer-related molecular targets of EGCG (in vitro studies).

Type of Cancer	Cancer Cell Line	Mechanism Involved	Reference
Breast cancer	MCF-7	↓Telomerase activity; ↓hTERT	[63]
Lung cancer	A549	↑G0/G1 phase arrest	[64]
Colon cancer	HCT116	↓TRAIL cell death	[65]
Esophageal cancer	Eca109 & Ec9706	↓Bcl-2 protein expression; ↑Bax and caspase-3 protein expression	[66]
Colorectal cancer	DLD-1 & SW480	↓Wnt/β-catenin pathway	[67]
Thyroid cancer	8505C	↓EMT by regulating the TGF-β1/Smad signaling pathways	[68]
Hepatocellular carcinoma	HCCLM6	↓MMP-2; ↓MMP-9; alteration in levels of FUBP1, HSPB1, CH60 and NPM proteins	[69]

Abbreviations used: TRAIL, tumor necrosis factor-related apoptosis-inducing ligand; EMT, epithelial to mesenchymal transition; TGF-β1, transforming growth factor beta 1; SMAD, SMAD family member; FUBP1, far upstream element (FUSE) binding protein 1; HSPB1, heat shock protein beta 1; hTERT, human telomerase reverse transcriptase; CH60, heat shock 60 kDa protein 1 (chaperonin); NPM, nucleophosmin; MMP, zinc-binding metalloproteinases. (↓) down/(↑) up regulation.

**Table 3:** Cancer-related molecular targets of EGCG (in vivo studies).

Type of Cancer	Study Model	Mechanism Involved	Reference
Pancreatic cancer	Balb/c mice	Inhibited tumor growth with ↓p-ERK, p-PI3K, p-AKT, and pFKHRL1/FOXO3a,	[70]
Hepatocellular carcinoma	Rats	↑DNA breakage; pro-oxidant effect	[54]
Breast cancer	Balb/c mice	↓VEGF	[71]
Colon cancer	BALB/c mice	↓HES1 and Notch2 induced the apoptosis	[72]
Skin cancer	ICR mice	↓TPA--induced DNA binding of NF--κB and CREB	[73]
Lung cancer	Mice	↓CSC-like characteristics by modulating the hsa-mir-485-5p/RXRα axis	[74]
Esophageal carcinoma	BALB/c mice	Induced apoptosis and ↑ROS generation, ↓VEGF; ↑caspase-3	[75]

Abbreviations used: CREB, cyclic AMP response element-binding protein; CSC, cancer stem cell; HES1, Hes Family BHLH Transcription Factor 1; VEGF, vascular endothelial growth factor; (NF-kB), nuclear factor kappa-B. (↓) down/(↑) up regulation.

## Data Availability

Not applicable.
